# Turning analysis into action: opportunities and challenges in implementing wastewater science for public health decision-making

**DOI:** 10.3389/fpubh.2025.1562659

**Published:** 2025-05-21

**Authors:** Anna Gitter, Valeria Ruvalcaba, Katelyn Clark, Theresa Tran Carapucci, Fuqing Wu, Blake M. Hanson, Jennifer Deegan, John Balliew, Eric Boerwinkle, Anthony W. Maresso, Kristina D. Mena

**Affiliations:** ^1^Department of Environmental and Occupational Health Sciences, UTHealth Houston School of Public Health, Houston, TX, United States; ^2^Texas Epidemic Public Health Institute (TEPHI), UTHealth Houston, Houston, TX, United States; ^3^Department of Epidemiology, UTHealth Houston School of Public Health, Houston, TX, United States; ^4^Department of Management, Policy and Community Health, UTHealth Houston School of Public Health, Houston, TX, United States; ^5^Department of Emergency Medicine, UTHealth Houston McGovern Medical School, Houston, TX, United States; ^6^UTHealth Houston, Houston, TX, United States; ^7^El Paso Water Utility, El Paso, TX, United States; ^8^TAILOR Labs, Baylor College of Medicine, Houston, TX, United States; ^9^Department of Molecular Virology and Microbiology, Baylor College of Medicine, Houston, TX, United States

**Keywords:** epidemiology, human health, viruses, quantitative microbial risk assessment, pandemic preparedness

## Abstract

In the 5 years since the emergence of the COVID-19 pandemic, the field of wastewater-based epidemiology (WBE) has dramatically expanded with programs implemented across the globe to monitor for SARS-CoV-2 and other viruses of public health concern. However, the best way to use wastewater surveillance data and inform local communities of the utility of wastewater science remains limited and sporadically discussed. Specifically, there is vague guidance regarding interpreting varying levels of viral loads in wastewater for public health significance. While collaborative efforts are key to implementing these community-specific wastewater surveillance programs, effectively using the data for public health decision-making still needs significant refinement. Aligned with recent calls for advancing the science of wastewater surveillance, the experiences described in this article examine the critical need to advance other aspects of WBE programs, including communication, ethics, and decision-making.

## 1 The evolving field of viral wastewater-based epidemiology

The focus of this perspective is to provide a follow-up on the recent advancements and challenges in wastewater science from our 2023 article (1). In the previous paper, the Texas Epidemic Public Health Institute (TEPHI) Wastewater Consortium (TWC) outlined steps, actions, and scientific methods to build a state-wide program for routine viral surveillance from wastewater sources. Here, we build upon the progress the TWC has made since its implementation in early 2022, and specifically highlight post-detection steps and considerations of data use that are important for a successful viral WBE program. This perspective includes discussion regarding the recruitment and participation of utilities in a WBE program, implementation and dissemination of the results for public health and communities, and the integration of WBE with risk assessment.

For a comprehensive review of the extensive literature of WBE and SARS-CoV-2 monitoring and research methodologies, the authors refer the reader to some excellent reviews on the topic (2, 3), including our previous 2023 article (1). Here, we provide a very brief summary highlighting WBE’s utility in viral tracking. In short, the COVID-19 pandemic reinvigorated viral WBE in early 2020 when several research groups across the globe reported the detection of SARS-CoV-2 in wastewater materials, in parallel with clinical cases. It is now well-established that the tracking of SARS-CoV-2 in wastewater is correlated to case numbers (4, 5), hospitalizations (6), community prevalence (7), waves of transmission (8), lead-indicator forecasting (9, 10), and variant detection (11–13). These findings have opened the door to targeted efforts to track viral spread in high-risk areas or populations, including on airplanes or in airports (14); cruise ships (15, 16); and in buildings (17), schools (18), nursing homes (19), and hospitals (20). The success of these efforts has prompted local and federal governments to implement systemized programs in many countries, including the U.S. (21), the European Union (22), Australia (23), and Israel (24). In some form or another, > 70 countries have indicated that viral wastewater monitoring is a part of their strategy to monitor SARS-CoV-2. The approach has been adapted to other respiratory viruses, including respiratory syncytial virus (RSV) (25), influenza (26), and measles (27).

Our own program began in April 2020 with SARS-CoV-2 and has since expanded to the wastewater virome (28), including H5N1 (29). The latter approach uses sequencing-based methods in addition to PCR, thereby utilizing an agnostic approach to attain viral information from > 3,000 different viruses, facilitating assessment of abundance, variant analysis, and spatial–temporal evolution, all in a single reaction. Since 2022, the program has sampled wastewater weekly and has now expanded to 15 Texas cities (38 total sites). A report of viral levels at each site for every detected virus is shared with local health departments and public health stakeholders across the state.

In this perspective, we share experiences, lessons learned, and future opportunities that have come from implementing this WBE program. We recognize that numerous WBE programs exist (21–24) and that there is a critical need to discuss how to disseminate, communicate, and utilize wastewater data beyond data collection. We hope to provide information and highlights from our program to strengthen the implementation of other WBE programs.

## 2 Community perspectives following the establishment of WBE programs

### 2.1 Public health considerations

The monitoring output from wastewater surveillance (including the identification and enumeration of microorganisms) can be used to inform of disease transmission within a sewershed, therefore helping to estimate the number of infections associated with specific pathogens circulating within a community (30). Collecting data through wastewater analyses is logistically more feasible than the clinical-based approach, wherein a public health department is alerted to a potential disease outbreak or case cluster of illnesses. For pathogens associated with nonspecific symptoms related to the gastrointestinal tract, the causative agent is typically never identified due to the absence of medical consultation and diagnostic testing. Such cases typically resolve on their own, yet can still lead to widespread transmission and therefore illnesses of the infectious agent. There is a critical need for public health practitioners to continue partnering with wastewater utilities to establish sewage sampling and analysis regimens. This partnership could help identify community demographics, vulnerable neighborhoods, and other factors that may inform which microorganisms to target and where to sample within a wastewater distribution system. For example, regions and cities with a larger proportion of older adult community members may want to prioritize opportunistic pathogens (31), just as rural areas will likely have different microbial agents of concern than urban areas. Local and national disease prevalence and vaccination rates should also be considered based on the pathogen of interest. Depending on available local resources, third-party laboratories may need to be contracted to aid in pathogen detection (whether targeted or agnostic). To optimize community resources, integrate diverse perspectives, and gather on-the-ground support, other stakeholders representing clinical practice, schools, and businesses should be involved in developing a community-level wastewater surveillance program.

Traditionally, case infections and illnesses are identified through clinical detection and diagnosis. Depending on the incubation period of the pathogen, wastewater surveillance data can be used to identify community infections sooner than a doctor visit can be scheduled (if scheduled at all) and earlier than infections can be noted through other syndromic measures, such as increased school absences or empty shelves at the pharmacy. This timely identification of the pathogen could lead to an early determination of the source of adverse health within a community [e.g., foodborne illness outbreaks (32)], informing mitigation approaches for public health practitioners. Characterizing the community wastewater microbial portfolio could highlight public health-relevant pathogens or potential pathogens of concern for clinicians. For example, a medical practitioner may not be familiar with or have clinical experience with the measles virus and, therefore, may not consider measles when seeing patients with fever, runny nose, and rash. Given the lengthy incubation period of this virus (up to 4 weeks) and its high likelihood of transmission, early community identification would be necessary to protect public health (33). Additionally, when patients present with non-specific symptoms, agnostic wastewater surveillance data can inform physicians to either directly test for a specific pathogen for a more efficient, cost-effective diagnosis or provide supporting information to treat a suspected (non-serious) infection. More broadly, such monitoring data can be used to identify infection trends that may impact medical resources, such as the number of available hospital beds, or forewarn of threats to particularly vulnerable sub-populations (such as the older adult) or eventgoers in mass gathering scenarios, including concerts and parades. Wastewater surveillance data can also provide public health meaning to other health indicators collected by a community’s health information exchange, with information about prevalence and incidence of certain diseases, vaccination, and insurance status throughout a region, or percentage of highly susceptible individuals (like pregnant women). Considering the devastating health and economic impacts of the COVID-19 pandemic, along with inconsistent testing and vaccination within populations, wastewater surveillance output of SARS-CoV-2 may have improved the content and interpretation of the mitigation guidance delivered. Wastewater surveillance programs, especially those with agnostic detection approaches, have the opportunity to provide valuable supplemental disease surveillance that is often lacking for most communities.

In May 2020, the TWC formed the Texas Wastewater and Environmental Biomonitoring (TexWEB) network (1). TexWEB’s mission, as part of the TWC, is to integrate advanced and innovative wastewater sequencing (agnostic pathogen detection) with every facet of the wastewater stakeholder continuum. As such, the group formed four workgroups; (i) Science and Validation, responsible for implementing the wet and computational lab efforts for detection; (ii) Statistics and Modeling, responsible for the creation of a dashboard and developing models for pathogen forecasting; (iii) Action Plan discussed below, but mainly to form a body of diverse expertise to weigh in and decide on essential monitoring matters; and (iv) the recently formed Ethics Workgroup, tasked with developing ways to share data, communicate, and protect community privacy and buy-in. The four workgroups address distinct domains of wastewater science to enhance the interpretation, dissemination, and impact of this data for public health.

### 2.2 Communication

The dissemination of wastewater surveillance and sequencing data to stakeholders and the broader community continues to evolve to meet community-specific applications. Visualization and communication of these datasets for local, state, and national decision-making is not yet standardized, let alone sharing these data for personal guidance. Further complicating this matter is the “interpretability” of the data for public health, given that the data can often not be assessed as a single point in time but requires context from prior concentration levels and trends (34). The TWC has been proactively collaborating to evaluate how best to disseminate information regarding viral loads in wastewater to the broader community. Survey results (unpublished), which included 34 respondents from public water utilities and public health departments across the State of Texas, indicated that the overwhelming majority are interested in receiving reports of recent wastewater surveillance results for viral pathogens, preferably weekly, with information for both statewide and local trends. The preferred form of information delivery included a public-facing, web-based dashboard with information regarding trends. However, respondents still identified the need to provide clear guidance regarding personal behavioral choices that can be made to meet individual risk needs. Ensuring that not only is data visualized and shared, but guidance regarding how to interpret the data is equally important.

The TWC’s communication strategy included implementing the Action Plan Workgroup, which consists of diverse stakeholders - local health departments, water utilities, clinicians, and participants from the Texas Department of State Health Services - to grapple with the challenges of articulating and disseminating wastewater data in a format that is applicable for public health action. Thus far, this work has included developing a weekly report that describes viral load trends in wastewater across all sites with participating wastewater treatment utilities, creating a public-facing dashboard that describes viral loads in wastewater and community health trends across all sampling sites, and collaborating with local health departments to develop their own public-facing dashboards to inform their constituents. However, regardless of the format and amount of information disseminated, it is critical that the program also provides actionable public health recommendations that accompany these platforms so that the data can drive decision-making. Due to the agnostic detection approach, TWC has consistently detected concerning viruses well before other surveillance measures (28), including the wastewater surveillance detection of avian flu H5N1 (29), which garnered media attention. As part of TWC’s mission, the data was reported to key members of the Action Plan Workgroup, and a decision was made to inform the local and state public health departments of the detection. Action Plan Workgroup leadership also served as a liaison to the CDC and White House in the communication and update of findings, activities which not only built trust within the stakeholder network but also led to focusing national attention on this viral threat (now monitored via wastewater).

### 2.3 Public concerns regarding the science

In light of the potential for practical application of wastewater surveillance to inform public health action, the science of the field has expanded to different viruses (1) while some experts have called for monitoring of other pathogens of human health concern (e.g., *mycobacterium tuberculosis*, *Candida auris*) and antimicrobial resistance. While wastewater surveillance has been widely reported and accepted as a valuable tool for monitoring trends over time, conveying community-level risk, and planning for healthcare utilization requires specific considerations which include: (a) the potential to identify individuals as genomic surveillance systems; (b) the possibility that data may be stigmatizing to specific communities; (c) lack of public education and awareness, creating a context in which misinformation can spread easily; (d) the potential to use wastewater surveillance for other purposes (e.g., law enforcement); and (e) fears that data could be used to justify unpopular public health interventions. We briefly address these public concerns with examples and the need to continue refining the science of wastewater surveillance.

In its current application, wastewater surveillance does not yield information used to identify individuals. Still, as science advances and data collection expands, sequencing whole human genomes from wastewater samples may become possible. Even in the context of current tools and applications, a recent study identified a rare SARS-CoV-2 variant with a probable single-source origin (35). Privacy concerns are magnified when the contributing catchment area comprises a relatively small number of households or individuals (e.g., congregated living settings, airplanes, small communities). However, community-level wastewater surveillance and related research are not regulated as human subjects research, and no consent is required of contributing individuals (36). There are no ethical guidelines to inform the collection, use, and dissemination of the data generated from wastewater surveillance, another reason for developing the Ethics workgroup in the TWC.

Additionally, there is the potential for the resulting data to stigmatize specific communities or groups. As interest grows in applying this science to sexually transmitted infections (STIs) (e.g., HIV, MPXV), caution should be exercised to ensure that surveillance methods and resulting public health action do not stigmatize or discriminate against certain groups and to engage potentially affected communities in developing safeguards (37). Despite the expansion of wastewater surveillance during and after the COVID-19 pandemic, there is a general lack of public awareness about the science and its capabilities (38). This dearth of understanding fosters an environment in which misinformation easily spreads. A significant example occurred in December 2023, when the news organization Breitbart posted on social media that high levels of COVID-19 had been detected in the nation’s water supply, leading some to speculate that the government was intentionally infecting the public through its drinking water (39). Efforts to expand the application of wastewater surveillance should be accompanied by community engagement and education efforts to ensure that reporting of results does not undermine public trust.

While there is general public support for wastewater surveillance to protect public health (38), other uses have the potential to weaken public support for the practice. In a recent report on wastewater surveillance to support public health action, the National Academies of Science, Engineering, and Medicine recommended that caution be exercised to guard against “function creep,” particularly regarding data use for law enforcement purposes (40). Finally, in the current context of the politicization of the nation’s response to the COVID-19 pandemic, concerns have been raised about the resulting public health actions taken to mitigate outbreaks. During the recent pandemic, actions such as mask and vaccine mandates and school closures were highly controversial and led some to surmise that public health practice was being used to advance a political agenda. Care should be taken to ensure that wastewater science does not become subject to similar controversy.

## 3 Implementation of the science for action

### 3.1 Connecting teams, utilities, and communities

**Figure 1:**
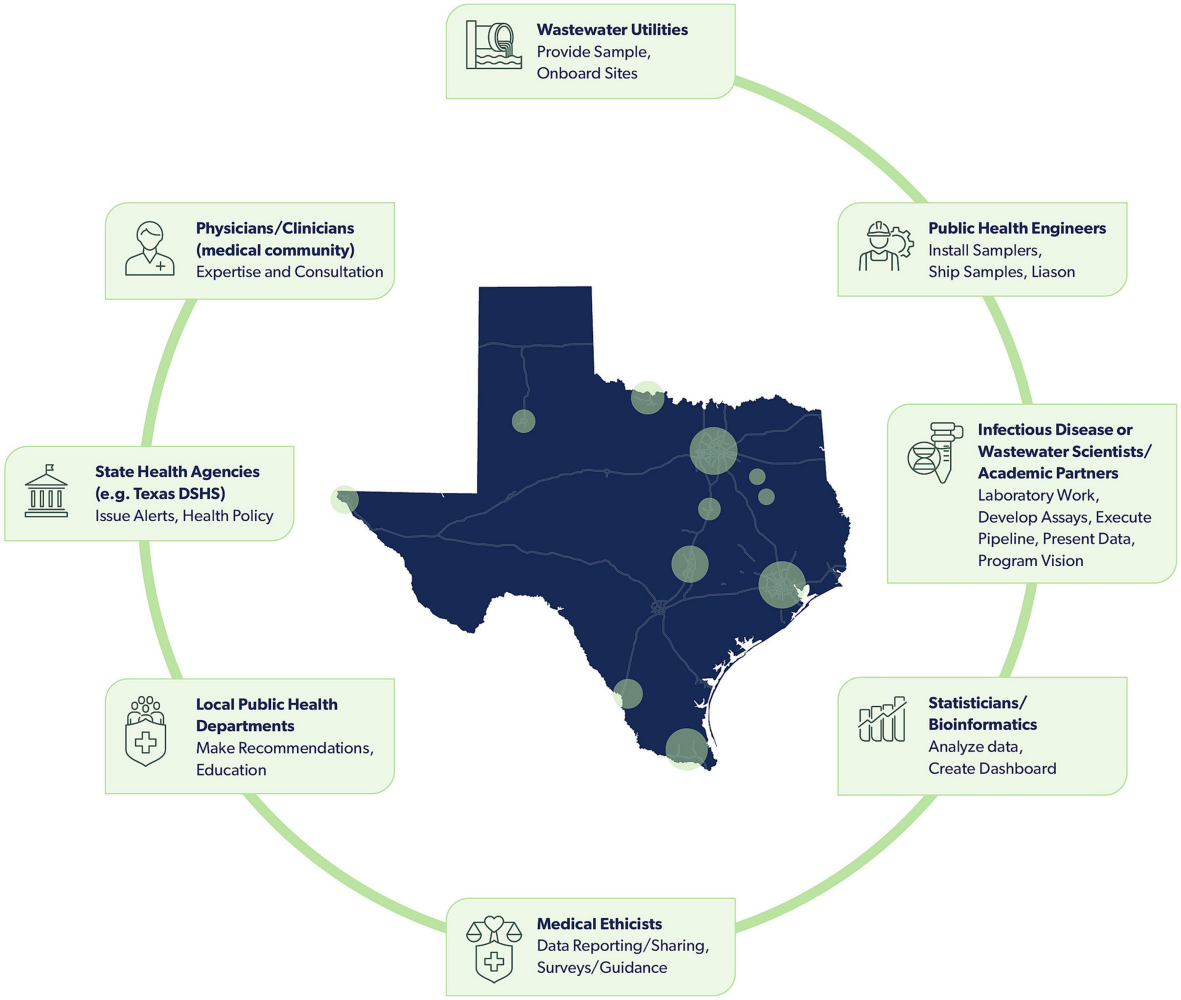
An array of diverse stakeholders support the TWC and TexWEB, with roles that include collecting, analyzing, evaluating, and disseminating wastewater surveillance data. Stakeholders, in sequential order of who participates in collecting and assessing wastewater data, include wastewater utilities, public health engineers, infectious disease or wastewater scientists/academic partners, statisticians/bioinformatics, medical ethicists, local public health departments, state health agencies, and physicians/clinicians. Circles present within the map of Texas signify cities with sites participating in the TexWEB network.

Successful implementation of a wastewater surveillance program requires engagement across disciplines and sectors, including utilities, public health departments, clinicians, data scientists, microbiologists, and epidemiologists. However, practitioners in this field do not have many, if any, natural venues for working together. Consequently, the TWC was formed to guide the efforts of the statewide TexWEB network and has representation from each of these sectors (Figure 1).

Two key challenges were identified early on in implementing the TexWEB network within TWC. First, recruiting and onboarding utilities required navigating legal agreements, costs associated with sampling and shipping, and even local political considerations that varied from one municipality to the next. Second, a process for reporting wastewater findings in a manner that supports timely and practical public health interventions had to be developed. The strategy to engage with local health departments and utilities required a two-pronged approach. The El Paso Water Utility, a TWC member well-versed in the challenges of wastewater surveillance from a utility’s perspective, conducted direct outreach and engagement with utilities across the state. These conversations were particularly critical in the early months of implementation during 2020 and 2021.

The Action Plan Workgroup began meeting in late 2022 to refine the list of viruses to be included in the targeted panel and develop notification and reporting protocols with the input of stakeholders charged with implementing public health interventions. To inform its efforts, the Action Plan Workgroup developed and administered a statewide survey of public health departments assessing interest in wastewater data and reporting preferences. Key considerations included report cadence, data granularity (e.g., statewide, local, by catchment area), audience (e.g., public reports versus reports to health departments), data representation (e.g., trend lines, heat maps), and reported pathogens. This work produced a report that is disseminated on a weekly basis to participating public health departments and was significantly informed by the perspectives of those working at the local level to implement public health action.

Additionally, the Action Plan Workgroup is building on this effort by informing the network’s geographic growth, expanding the detection of non-viral pathogens, and implementing some public data reporting (e.g., seasonal respiratory viruses such as RSV, influenza, and SARS-CoV-2). The group is also advising on the development of a tabletop exercise to help local health departments refine policies and procedures triggered by the detection of a viral signal in wastewater. Future efforts should also encompass stakeholder engagement to identify and develop the best practices in ethical reporting of wastewater data.

### 3.2 Medical community engagement

Medical providers are essential liaisons between public health entities and the community being served. They are positioned to intimately understand their practice populations during visits and determine population-level trends via electronic medical record databases. Though it has become more common for physicians to consider public health topics such as non-medical drivers of health and health equity in their care delivery, the medical community and public health sector still face communication challenges. This problem was highlighted at the beginning of the COVID-19 pandemic, when providers became suddenly overwhelmed with sick patients, while the U.S. public health system lagged in identifying, testing, tracing, and controlling the spread of the virus (41). Exacerbating the problem, physicians were sometimes the culprits in spreading misinformation during the “infodemic” that accompanied the pandemic, resulting in confusion, mistrust of the scientific community, and worse health outcomes (42–44).

For nearly all medical issues, individual patients and practice communities seek the guidance of their medical providers, and consequently, these medical providers are often the “face” of the public health response system. Since physicians are considered the most trusted source of health information (45), it is critical that public health programs, such as a WBE program, work closely with them to educate the community (46). A WBE program should engage physicians early in the implementation process to determine the most useful and relevant information for medical providers to deliver the best preventive and responsive medical care. In the case of respiratory illnesses, different diseases may present as similar syndromes, but treatment options differ depending on the diagnosis. In many settings, testing for specific diagnoses using a full respiratory test panel is time - and cost-prohibitive, leading to the physician treating the most likely disease at that time. The “most likely disease” may be determined by personal gestalt and experience, memos from health care administrators, or discussions amongst colleagues. A WBE program that can alert the medical community of a specific impending endemic would improve physicians’ pre-test probability of choosing the correct treatment pathway for their patients, their likelihood of testing for specific diseases, and their delivery of patient education. It would also allow health systems to prepare for imminent surges in hospitalization and allow pharmacies to anticipate increased demand for specific medications (47, 48).

However, there is still much ground to gain regarding informing the medical community of existing WBE programs. A recent questionnaire administered to infectious disease physicians by the Centers for Disease Control and the Infectious Diseases Society of America regarding wastewater surveillance revealed that only 22% of respondents reviewed wastewater surveillance data, with most individuals not reviewing data regularly or knowing if data was available (46). Nearly all states implement a WBE program, but there is still a gap regarding knowledge or public access to these data. Key information that a WBE program could provide to the medical community includes the general location, local prevalence, rate of spread, transmissibility, anticipated emergence of clinical illness, and potential duration of an endemic disease. If paired with clinical guidelines and maintained in a user-friendly and reliable dashboard accessible to health care providers, this information would be invaluable to physicians during their point-of-care treatment decisions.

## 4 Expanding the utility of WBE programs

### 4.1 Interpreting viral loads in wastewater for public health risks

While WBE programs, much like TexWEB (within the TWC), have advanced in their robustness of molecular and metagenomic analyses, there remains a critical gap in how to interpret the wealth of data that continues to be collected weekly or even daily. Thresholds or indices for varying levels of viral load in wastewater are not uniformly applied across WBE programs, and site-specific approaches developed by local researchers and public health practitioners are primarily the programs being implemented at this time. Consequently, there are differing metrics and perceptions regarding tolerable thresholds for endemic viruses in wastewater, with an imperative underlying recognition for community-specific factors to drive the need for varying thresholds.

Quantitative and qualitative thresholds have been proposed to assess wastewater viral loads and their associated health risk levels. Other communities have developed qualitative indices, such as the Wastewater Viral Load Risk Index, to interpret their wastewater data, developing a four-category risk framework – low, medium, medium-high, and high – that utilizes assumptions regarding the virus reproduction number, daily per capita concentrations of virus in wastewater, clinical data, and weekly viral load change rate (49). A more quantitative approach has been undertaken to utilize SARS-CoV-2 viral loads in wastewater to infer total infection levels in a community (30). Mechanistic models utilizing several parameters, including daily flow, per-capita wastewater generated, endogenous microbial markers, and SARS-CoV-2 RNA signals, have attempted to estimate infection counts for specific communities. However, many factors continue to hinder the utility of a defined community-specific viral threshold in wastewater for SARS-CoV-2 and other pathogens due to the complexity of sampling strategies (50), array of different molecular analyses for quantification (51, 52), sewer biofilms (53, 54), fecal shedding rates (55–57), and pathogen decay rates in wastewater (58).

Tolerable risk is an accepted approach by the U.S. Environmental Protection Agency when evaluating drinking water (59, 60) and recreational waters (61) and can potentially be applied to wastewater science. A potential tool to inform of tolerable viral loads of SARS-CoV-2 in wastewater is quantitative microbial risk assessment (QMRA). This risk assessment framework has been extensively used to assess environmental exposures to pathogenic microorganisms and associated health risks (62, 63), and more recently has been utilized to assess potential exposure to SARS-CoV-2 in bioaerosols and incidental water ingestion by wastewater treatment plant workers (64–66). Specifically, a reverse-QMRA approach is being developed by the TWC to interpret wastewater viral loads in the context of estimated infection levels, and ultimately, to be able to propose a threshold viral load for community infections based on a tolerable risk level. Additionally, the framework for this work would employ a mechanistic model that incorporates several parameters, including shedding rates, viral decay, recovery, dose–response model for infectivity, and morbidity, to account for inherent variability and uncertainty, thereby strengthening the application of the model to guide public health decision-making.

### 4.2 Disease X and viral preparedness

The World Health Organization has included the concept of Disease X in its pandemic blueprint of diseases for which research and monitoring would be of international importance (67). Disease X refers to the next pathogen, currently unnamed, that may evolve or zoonotically emerge that will cause mass morbidity and mortality to humanity (68). Although there are several simulations and table-top exercises that international governments or organizations routinely perform to prepare for pandemics, they do not generally involve downstream action following the detection of a pathogen in wastewater material. When they do, guidance relates to what the utility will (or should) do to safeguard water quality and water operations, but not how information attained about pathogens in wastewater can be used to prepare society for a coming epi- or pandemic (69, 70). This leaves an obvious question: Given the interest in wastewater analysis for public health action, the emergence of national programs, and the application of cutting-edge technology, which makes sampling, detection, and analysis higher throughput and more cost-effective, how can the massive amount of information about virus levels be used to make targeted, wide, and actionable public health interventions?

**Figure 2:**
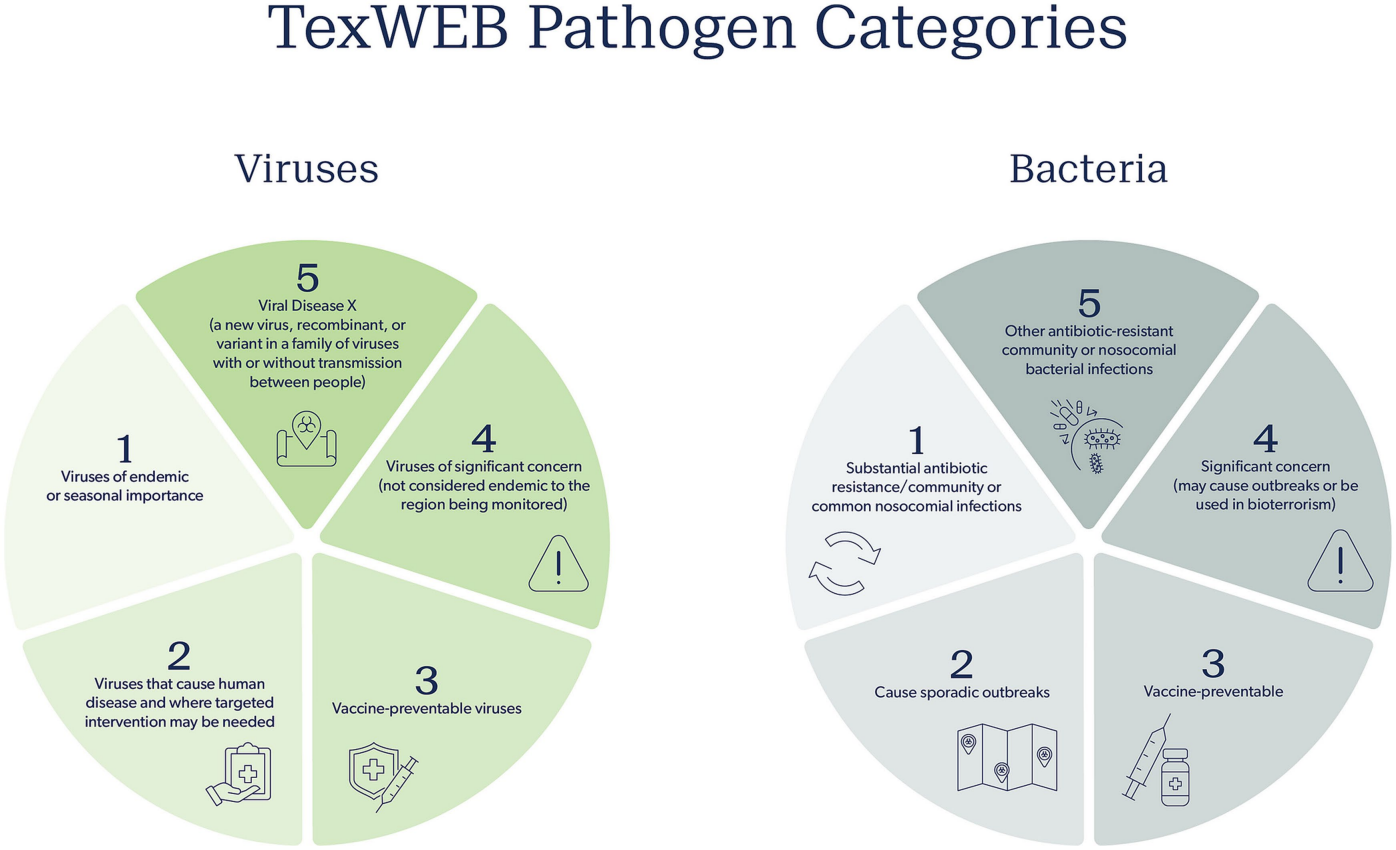
TWC pathogen categories for both viruses and bacteria to inform appropriate public health response and action. Five categories are proposed that outline the nature of the pathogen, increasing in public health concern from category one to five.

To address this question, the TWC proposes categorizing viral WBE data into five main categories, separated by type of virus and its interest to community public health or the research community (Figure 2). Category 1 includes viruses of endemic or seasonal importance. Respiratory viruses in this category include influenza, RSV, SARS-CoV-2, Enterovirus D68, Human metapneumovirus, and Parainfluenza viruses, as well as all their variants and quasi-species. WBE may be very useful in being a lead indicator or orthogonal validation of the seasonality or outbreaks of these viruses, especially since all have been detected in wastewater. Category 2 includes viruses that may cause human disease but have more sporadic transmission patterns. Here, targeted intervention may be necessary if the signal reaches a certain threshold, is prolonged, and is validated by an increase in cases, but for the most part, this category is associated with viruses for which there are no formalized efforts to intervene. This includes several enteric viruses such as norovirus, adenoviruses, and bocaviruses, all three of which are routinely detected in our sampling efforts, though we are currently uncertain what global health effects they are having on populations. Category 3 can be considered vaccine-preventable viruses, such as those that cause measles, smallpox, rabies, and polio. Should a signal be observed for these viruses when historically there were none, it may mean vaccine coverage is falling, a sign that campaigns to increase the vaccination prevalence against these preventable infections are warranted. Category 4 includes viruses of catastrophic consequence, such as hemorrhagic viruses (e.g., Ebola, Hanta) or viruses of arthropod-borne origins (such as many of the arboviruses – West Nile, Zika, Chikungunya, and Dengue). These viruses tend not to be endemically sustained in the region being surveyed and are not considered a novel disease or variant of concern. But if detected, these viruses warrant immediate laboratory validation and downstream investigation. At the very least, the public health network should be notified to put the signal on their radar should clinicians start seeing patients with symptoms that may be related to these infectious viruses. Finally, category 5 consists of a viral Disease X, a new virus, recombinant, or variant in a family of viruses whose presence is a concerning sign, with or without transmission between people. The emergence of H5N1 is a good example, as is a variant of SARS-CoV-2 for which the vaccine is ineffective.

The potential responses for each category vary significantly. The TWC views category 1 viruses with WBE as amenable to a weekly weather report. Dashboards indicate a change in their abundance, and certain precautions are forecasted for a coming season or period of time based on what is circulating. The “triple epidemic” of flu, COVID-19, and RSV in 2022 is an example of this. In these scenarios, public health officials would disseminate alerts that the seasonal respiratory illness season has started, vaccination campaigns can be accelerated, and hospital staff can be put on notice that admissions are likely to rise. For category 2 viruses detected by WBE, the context is important. A sudden spike of norovirus may mean an outbreak in a nursing home or school. For this, the general public may not need a notice, but public health officials may investigate locally for a more targeted intervention. For category 3, a sudden and sustained rise in a vaccine preventable virus (measles, for example), after laboratory validation, should be used by health authorities to conduct clinician education and public outreach about the necessity of routine vaccination for preventing outbreaks, especially amongst children, since such outbreaks are often attributed to anti-vaccine or low vaccine uptake in specific communities (71). Responses for category 4 and 5 viruses detected via WBE are more nuanced. A signal in wastewater from a hemorrhagic virus would be of grave concern, but it might also cause unnecessary panic amongst the public and health authorities if not properly validated. However, if validated, health authorities should be informed and put on immediate alert, using this information to inform nearby hospitals and health care networks to add diseases caused by such viruses to their clinical radar. In this situation, the development of a standard operating procedure and risk assessment is of most value for viral WBE. Using existing procedures, such as those from the BioWatch select agent air monitoring program, can guide the development of a WBE action policy (72). At the moment, the TWC has an active R&D effort to develop the bacterial version of the total virome approach that is the current thrust of the program. Much like the virome, the “bacterialome” aims to agnostically report on nearly every bacterial species known to be of significant concern for public health. The effort will focus on tracking antibiotic resistance and bacterial pathogens of concern, mostly from an outbreak standpoint. Building on the success of using probe capture to identify stretches of bacterial nucleic acid and short-read sequencing, the TWC will focus on five defining categories of pathogenic bacteria (Figure 2). Category 1 involves the so-called ESKAPE bacteria, which are responsible for the most antibiotic-resistant infections in the community and hospitals. Here, not only will the species be tracked, but, of great value, the genetic elements that confer drug resistance and virulence will be tracked, thereby providing a comprehensive picture of the two genomic factors that primarily drive bacterial diseases. Category 2 will focus on bacteria that frequently cause outbreaks, often foodborne in nature. Category 3 will monitor pathogens in unvaccinated children. This category is especially important to monitor as concerns rise about lowered childhood vaccination rates. Category 4 includes pathogens of substantial virulence and concern, mostly species that should not be present or readily reported in the community unless there is an outbreak or malicious intent. Finally, category 5 includes oft-ignored non-ESKAPE drivers of drug resistance, which are commonly reported in hospital settings. Whereas it is unclear how knowledge of these bacterial pathogens and their presence will be used, they would certainly be a public health burden due to knowing very little about their prevalence, transmission rates, and distribution in space and time among populations.

## 5 Next steps and future directions

While the scientific robustness of wastewater surveillance continues to advance with new possibilities, so does the need for appropriate interpretation of the data for decision-making and the engagement of diverse stakeholders to enhance the value of these programs. It is critical that the scope of a wastewater surveillance program extends beyond that of research to include guidelines that require using these data for public benefit. The TWC recognized this need for diverse stakeholders to identify actionable items and formed workgroups to address preparedness and communication needs. This work should not be siloed within the realm of public health, medical, and water utility stakeholders. These programs, which can identify proactive measures and early warnings for community disease transmission that are beneficial for public health, can also inform workplace safeguards to ensure businesses stay open. The agricultural sector, which is faced with securing the safety of our produce, faces challenging tasks in ensuring source water quality to mitigate pathogens associated with foodborne illnesses. Large employers may need to be aware of disease trends to anticipate upcoming periods of reduced staff availability. If there is anything we learned from the COVID-19 pandemic, it is that infectious disease pathogens affect all facets of our daily life. Wastewater surveillance is already informing decision-making for medical and public health communities, and tools used in these programs may also assist other sectors.

There is also the opportunity not to operate WBE programs in silo, and to collaborate at local, state, national, and international levels to share scientific methodologies, wastewater data, and public health implementation strategies. To mitigate the next major outbreak, epidemic, or even pandemic, data must be shared, and collaboration across political jurisdictions can help meet pandemic preparedness needs. These collaborations will also strengthen public health surveillance networks, improving overall community and population health.

We propose a few actionable recommendations that we hope other wastewater surveillance programs can implement to enhance the utility and direct impact of their work. These recommendations include developing specific workgroups to improve the science and application of the program. For example, consider forming a science validation committee to conduct additional analyses to ensure positive results for specific pathogens (e.g., measles, H5N1) are indeed valid if detected. An Action Plan Workgroup provides an opportunity to engage with community stakeholders to ensure that the data collected is disseminated appropriately and impactfully while also building community support for your program. We recommend engaging beyond public health departments, but with the greater medical community, including emergency and primary care physicians. These stakeholders should be included in workgroups or community networks if desired. Lastly, it is essential to establish a response framework to inform of actions and communication channels if a positive detection (that is of significant public health concern) occurs. We hope the categories we described help guide response practices for other wastewater surveillance programs.

The TWC viral WBE program, TexWEB, has been conducting routine sampling across several sites in Texas for over 3 years and continues to improve the communication and translation of these data for diverse stakeholders. Future work should also include developing educational training platforms to prepare the next generation of wastewater scientists to continue this work locally, nationally, and internationally. As more data and stakeholders are included, we plan to report new lessons learned in hopes of helping other WBE programs succeed.

## Data Availability

The original contributions presented in the study are included in the article, further inquiries can be directed to the corresponding authors.
